# A ten-year, single-center experience: Concordance between breast core needle biopsy/vacuum-assisted biopsy and postoperative histopathology in B3 and B5a cases

**DOI:** 10.1371/journal.pone.0233574

**Published:** 2020-05-21

**Authors:** Mohamed Elsharkawy, Thomas Vestring, Hans-Juergen Raatschen

**Affiliations:** 1 Diagnostic and Interventional Radiology/Neuroradiology Department, Agaplesion Diakonieklinikum, Rotenburg Wuemme, Germany; 2 Diagnostic and Interventional Radiology Department, Hannover Medical School, Hanover, Germany; University of Toronto, CANADA

## Abstract

**Purpose:**

To determine the concordance rate between core needle biopsy/vacuum-assisted biopsy (CNB/VAB) and postoperative histopathology in B3 (lesions of uncertain malignant potential) and B5a (in situ) lesions found on mammograms or ultrasound.

**Material and methods:**

2,029 consecutive biopsies performed over 10 years for patients who underwent mammograms or ultrasounds. For CNB 14G needle and for VAB 8G/10G needles were used. In all biopsies, we identified the age, BI-RADS®, histopathological biopsy results, B-category, nuclear grade for DCIS and postoperative histopathology results in B3 and B5a cases from the biopsy.

**Results:**

The B-categories from CNB/VAB were as follows: B2 42.2 percent (n = 856), B3 4.5 percent (n = 91), B5a 5.7 percent (n = 115), and B5b 47.6 percent (n = 967). In the B3-category, 72/91 patients underwent surgical excision, with a concordance rate of 83.3 percent (n = 60/72) and a discordance rate of 16.7 percent (n = 12/72) to postoperative histopathology. From the discordant cases, 67.7 percent (n = 8/12) showed DCIS and 32.3 percent (n = 4/12) showed invasive breast cancer. The BIRADS of the discordant cases was 4b in 41.7 percent (n = 5/12) and 5 in 58.3 percent (n = 7/12). The PPVs for malignancy of B3 lesions were 0.21, with no statistical significance between subgroups. In the B5a-category, 101 of 115 patients underwent surgery in our hospital, with a concordance rate of 80.2 percent (n = 81/101) and a discordance rate of 19.8 percent (n = 20/101) to postoperative histopathology. From the discordant cases, 55 percent (n = 11/20) showed invasive breast carcinoma of no special type (NST).

**Conclusion:**

Our concordance rate for B3 (83.3 percent) and B5a (80.2 percent) lesions in the biopsies to postoperative histopathology is matching to previously published literature. Surgical excision is our recommendation for lesions biopsied with a B3 category in the histopathology and a BIRADS category of (4b, 4c and 5). The PPVs for malignancy of B3 lesions showed no statistical significance between subgroups. Also, the nuclear grade of DCIS was not statistically significant in terms of upgrade into invasive breast cancer.

## Introduction

In Europe, the female breast was the most common cancer site in 2018 (523,000 cases) [1]. Breast biopsies are commonly performed to evaluate mammographic or palpable findings that are of concern, and the majority reveal benign findings [2].

The breast imaging and data system (BI-RADS®) categorizes mammographic findings from 0 to 6 [3], with categories 4 (including a- low; b- moderate; and c- high suspicion of malignancy) and 5 requiring tissue biopsy. According to guidelines for non-operative diagnostic procedures and reporting in breast cancer screening, the histological results of core needle biopsy (CNB) and vacuum-assisted biopsy (VAB) are categorized from B1 to B5 [4]. The B1 and B2 categories respectively represent normal and benign lesions, while the B4 and B5 categories respectively represent suspicious and malignant lesions [4]. The B3 category comprise lesions with uncertain malignant potential. The B5 category is further subdivided into B5a, which comprise in-situ carcinomas and the B5b, which comprise the invasive carcinomas [4].

The B3 category represents a heterogeneous group of lesions (for example: atypical ductal hyperplasia, radial scar, papillary lesions, etc.) that may be associated with malignant disease requiring surgical intervention [5]. With the increasing use of mammographic screening, the detection rate of B3 lesions in patients who were previously asymptomatic has increased [6, 7]. This has resulted in breast surgery for ultimately benign final histopathological diagnoses [5]. The rate of B3 lesions in biopsies ranged from 3.8 percent to 9.2 percent in screening program or single institution [8–13]. El Sayed et.al found that B3 rate was 5 percent from all CNBs (20.001) in screening program over 10 years [14]. Weigel et al, reported a B3 rate of 15.1 percent in digital mammography screening over a period of 4 years [15]. Lee et al, found that the B3 rate was higher in screening program compared to symptomatic patients (7.3 percent vs 2 percent) [6].

Furthermore, ductal carcinoma in-situ (DCIS), is primarily diagnosed by imaging because it is usually clinically occult [16]. DCIS was previously an uncommonly identified breast lesion, now it accounts for approximately 20 percent of newly diagnosed breast cancer cases [17]. As the detection rate increased, there has also been increased discussion and controversy [18]. Because of the heterogenous nature of DCIS, the disease process is in part not well understood [19]. Although it sometimes presents as a nonaggressive occult lesion, untreated DCIS may progress into an aggressive, invasive cancer [20, 21].

El Sayed et al, found that underestimation rate of malignancy associated with B3 lesions is 19.1% [14] and Brennan meta-analysis showed that approximately one in four DCIS diagnoses by CNB represents understaged, invasive breast cancer [22].

Preoperative variables significantly associated with understaging include: Biopsy device and guidance method; size; grade; mammographic features; and palpability [22].

Therefore, we focused on lesions of unknown malignant potential and in-situ carcinomas in our single-center study. This paper will elucidate the concordance rate between tissue biopsies and postoperative histopathology over the course of 10 years. Imaging and pathological findings of the biopsies are concordant when the pathology results adequately explain the imaging features. If the pathological results do not adequately explain the imaging features the two are considered discordant [7].

## Materials and methods

This retrospective study was performed after approval from the Ethics committee of Hannover Medical School. Given the retrospective nature of the study and data anonymization additional consent was waived by the ethics committee. Written informed consent was obtained from each patient before the biopsy was performed. Our retrospective study included 2,029 consecutive biopsies for patients who underwent mammograms or ultrasounds at our Hospital from January 2006 to December 2015.

Mammograms were done using a Mammomat® 3000 Nova (Siemens Healthcare, Erlangen, Germany), combined with a Fuji FCR 5000 MA reader (Fuji Medical Systems, Tokyo, Japan) from 2006 to 2010; and a flat-panel mammography with Mammomat Inspiration (Siemens Healthcare, Erlangen, Germany) from 2011 to 2015. All CNBs were performed using a coaxial, 14-gauge cutting needle with 15 mm or 22 mm penetration depths (Bard® MAGNUM® Biopsy System) by freehand sonography, using an Elegra scanner in 2006 (Siemens Healthcare, Erlangen, Germany); a LogiQ S6 scanner from 2007 to 2012 (GE Healthcare, Chicago); and an Acuson S2000 scanner from 2013 to 2015 (Siemens Healthcare, Erlangen, Germany). From 2006 to 2010 all VABs were performed using a 10-guage needle (VACORA® Breast Biopsy System, Bard®), under stereotactic guidance using mammography. From 2011 to 2015, VABs were done using 8-guage needle (Mammotome ST Biopsy Device) and a Mammotest table (Siemens Healthcare, Erlangen, Germany).

For each case, we collected the following data: age, BI-RADS®; guidance; histopathological biopsy results; B-category; DCIS nuclear grade, re-biopsy rate and postoperative histopathology results in B3 and B5a cases from the biopsy.

We specially focused on the concordance rate of the biopsy results compared to postoperative histopathology for patients with B3 and B5a category results in their biopsies. A review of the diagnostic mammograms and ultrasounds of the underestimated lesions in both categories was also done.

### Statistical analysis

The data was analyzed using the IBM SPSS software package, V. 20.0. (Armonk, NY: IBM Corp). Kolmogorov-Smirnov, Shapiro and D’agstino tests were used to verify the normality of variable distribution. Comparisons between groups for categorical variables were assessed using chi-square test (Fisher’s exact test). Significance of the obtained results was documented at the 5 percent level.

## Results

A total of 301 Vacuum assisted biopsies were performed under stereotactic guidance and 1728 core needle biopsies under ultrasound guidance were performed. Distribution of the B-categories, age and guidance are summarized in Table 1.

**Table 1:** Distribution of the different B-categories.

B-Classification	B2	B3	B5a	B5b	Total (n)
Age range in years	16–94	20.9–87.8	33.9–85.8	20.6–96.5	
Mean Age SD	51.46 ± 14.14	56.33 ± 13.67	59.24 ± 12.69	63.29 ± 14.37	
Guidance					
Ultrasound	711	63	27	927	1728
Stereotactic	145	28	88	40	301
Total (n)	856	91	115	967	2029

Seventy-three lesions were re-biopsied due to discordance between imaging and histopathological results of the VAB/CNB biopsies. From the 73 biopsies, 10 lesions histopathology results changed from B2 to B5b,1 lesion changed from B2 to B5a, 4 changed from B1 to B2 and 58 lesions did not change with a B2 result in both biopsies. B4 category was not included because those were only found in preliminary pathology reports of 4 cases which was categorized as B5a in the final histopathology report of the biopsy.

For the lesions with uncertain malignant potential (B3) and in-situ carcinoma (B5a) with CNB or VAB histopathology, a comparison to postoperative histopathology was done to show the concordance rate.

In the B3 category, 72 of 91 patients underwent surgical excision in our hospital. From the 19 non operated patients 12 had mammogram or ultrasound follow-ups for at least one year with no radiologic changes requiring a re-biopsy or surgical excision. Seven patients did not come for follow-up after the biopsy result.

Concordance between CNB/VAB and postoperative histopathology for B3 was found in 83.3 percent (n = 60/72 patients) cases, and discordance was documented in 16.7 percent (n = 12/72 patients) of cases. Three patients had two lesions, i.e. a total of 75 lesions. The 12 cases that were discordant with histopathology were upgraded as follows: Eight patients with DCIS (B5a) and four with invasive breast cancer (B5b). Table 2 summarizes the discordant cases regarding B3 lesions in CNB/VAB histopathology, compared to postoperative histopathology.

**Table 2:** Pre- and postoperative histopathology in discordant cases from the B3 category.

CNB/VAB histopathology vs. postoperative histopathology	DCIS G1 (n)	DCIS G2 (n)	DCIS G3 (n)	Poorly differentiated carcinoma (n)	Tubularadeno CA (n)	NST- LCIS- Lobular CA (n)	DCIS G2- NST- LCIS (n)	Total number
ADH (n)	1		1			1		3
ADH+PL (n)	1	1	1					3
PL (n)	1	1	1					3
LN (n)					1			1
FEA (n)				1				1
FEA+LN (n)							1	1
Total number	3	2	3	1	1	1	1	12

ADH = atypical ductal hyperplasia; PL = papillary lesion; LN = non-invasive lobular neoplasia; FEA = flat epithelial atypia; DCIS = ductal carcinoma in-situ; NST = invasive breast carcinoma of no special type; LCIS = lobular carcinoma in-situ; CA = carcinoma; (n) number of patients.

The most frequently excised lesion in the B3 subgroups was papillary lesion (50.7 percent), followed by atypical ductal hyperplasia (20 percent), flat epithelial atypia (12 percent), lobular intraepithelial neoplasia (8 percent), radial scar (5.3 percent), and phyllodes tumor (4 percent). Table 3 summarizes the frequency of the different B3 lesions from the biopsies and the rate of malignant diagnosis after excision.

**Table 3:** Frequency of the different B3 lesions from the biopsies and the rate of malignant diagnosis after excision.

B3 lesion	Frequency	Malignant diagnosis after excision	PPV	χ^2^	p
LN	6	2	0.33	0.560	^FE^p = 0.602
ADH	15	6	0.40	3.893	^FE^p = 0.075
PL	38	6	0.15	1.411	0.235
FEA	9	2	0.22	0.005	^FE^p = 1.000
RS	4	0	0	1.146	^FE^p = 0.572
PT	3	0	0	0.847	^FE^p = 1.000
Total	75	16	0.21		

χ^2^: Chi square test FE: Fisher Exact p: p value for comparing between the studied groups

LN = non-invasive lobular neoplasia; ADH = atypical ductal hyperplasia; PL = Papillary lesion; FEA = flat epithelial atypia; RS = radial scar; PT = phyllodes tumor

From the underestimated lesions in B3 category in our study, 83.3 percent (n = 10/12) of the lesions were identified on mammograms, 66.6 percent (n = 8/12) were identified on ultrasound and 50 percent (n = 6/12) identified on both.

On the mammograms 41.6 percent showed a mass (n = 5/12), 50 percent microcalcifications (n = 6/12) and 25 percent architectural distortion (n = 3/12). On the Ultrasounds 50 percent showed a mass (n = 6/12), 16.7 percent architectural distortion(n = 2/12) and 8.3 percent a hypervascular area (n = 1/12). From the 38 papillary lesions excised we had 32 intraductal papillomas without atypia (associated for example, with columnar metaplasia, sclerosing adenosis or usual ductal hyperplasia), 2 intraductal papillomas with atypia, 3 intraductal papillomas associated with ADH and one with multiple peripheral intraductal papillomas. The upgraded papillary lesions (n = 6) presented 3 times as a mass forming lesion, 1 time as a hypervascular mass, 1 time with calcification, and 1 time as architectural disturbance.

In the B5a category, 101 of 115 patients underwent surgeries in our hospital (14 patients with DCIS in CNB/VAB, with no postoperative histopathology report in our records). Concordance rate was 80.2 percent (n = 81) and discordance rate was 19.8 percent (n = 20). High grade DCIS was recorded in 68 cases, from those 17 upgraded to invasive breast cancer.

Intermediate grade DCIS was recorded in 19 cases, from those 3 upgraded to invasive breast cancer. Low grade DCIS was recorded in 14 cases, from those non upgraded to invasive breast cancer.

The upgraded invasive carcinoma types were as follows: Eleven cases with invasive breast carcinoma of no-special type (NST); two cases with invasive lobular carcinoma; two cases with micro-invasion; one case with invasive lobular carcinoma and Paget´s disease of the nipple; one case with invasive breast carcinoma of no-special type (NST) and mucinous carcinoma; one case with tubular carcinoma; one case with papillary carcinoma; and one case with poorly differentiated carcinoma. The discordant cases from the B5a category are summarized in Table 4.

**Table 4:** Pre- and postoperative histopathology in discordant cases from the B5a category.

postoperative histopathology vs. CNB/VAB histopathology	DCIS Intermediate grade (n = 3)	DCIS high grade(n = 17)	Total
NST	2	9	11
Invasive		2	2
Lobular			
carcinoma			
Micro-Invasion		2	2
Invasive		1	1
Lobular carcinoma,			
Paget´s disease			
NST and Mucinous carcinoma		1	1
Tubular carcinoma	1		1
Papillary carcinoma		1	1
Poor differentiated carcinoma		1	1
Total (n)	3	17	20

From the underestimated lesions in B5a category in our study, 85 percent (n = 17/20) of the lesions were identified on mammograms, 55 percent (n = 11/20) were identified on ultrasound and 40 percent (n = 8/20) identified on both.

On the mammograms 25 percent showed a mass (n = 5/20), 60 percent microcalcifications (n = 12/20), 5 percent architectural distortion(n = 1/20). On the Ultrasounds 50 percent showed a mass (n = 10/20), 5 percent a hypervascular area (n = 1/20) and none showed architectural distortion.

The biopsy needle size was documented for all lesions from the B3 and B5a category operated in our hospital to show the underestimation rates in each of the 8G, 10G and 14G needles used. There was no significant difference regarding smaller needle diameter and underestimation of malignancy, because of the overall small number of patients in both B-categories. Table 5 shows the results.

**Table 5:** Underestimation rates of the biopsy devices used.

B-category/Needle	B3 (n = 72)	Underestimation rate	B5a(n = 101)	Underestimation rate	Total underestimation
8G	10	2 (20%)	28	3(10.7%)	5/38 (13.1%)
10G	10	3 (30%)	49	8 (16.3%)	11/59 (18.6%)
14G	52	7(13.4%)	24	9 (37.5%)	16/76 (21%)

The most frequent histological diagnosis in the B2 category was fibroadenoma (29.9 percent), followed by Fibrous-, fibrocystic change (24.9 percent), sclerosing adenosis (16.1 percent), mastitis (7.4 percent), fat necrosis (7.3 percent), usual ductal hyperplasia (5 percent), simple cyst (4.7 percent), abscess (1.2 percent), and then other lesions (3.5 percent) such as lymph node, hematoma, pseudocyst and lipoma.

The most frequent histological diagnosis in the B5b category was invasive breast carcinoma of no-special type (87 percent), followed by lobular invasive carcinoma (9.7 percent), mucinous carcinoma (2.5 percent), tubular carcinoma (0.5 percent), and then other carcinomas (0.3 percent).

### Radiological/histological correlation

This was done using BI-RADS and B-category of the biopsy, the results are summarized in Table 6. Then we also calculated the results for the underestimated lesions and its effect on this correlation.

**Table 6:** Correlation of BI-RADS categories to B-categories for all biopsies.

BI-RADS vs. B-categories	B2 (42.2%)		B3 (4.5%)		B5a (5.7%)		B5b (47.6%)		Total	
	No.	%	No.	%	No.	%	No.	%	No.	%
4a	504	58.9	43	47.3	13	11.3	32	3.3	592	29.2
4b	329	38.4	37	40.7	70	60.9	323	33.4	759	37.4
4c	9	1.1	3	3.3	19	16.5	105	10.9	136	6.7
5	13	1.5	8	8.8	13	11.3	503	52.0	537	26.5
6	1	0.1	0	0.0	0	0.0	4	0.4	5	0.2
Total	856		91		115		967		2029	

In the BI-RADS 4a Category 547 lesions had B2 and B3 results in the biopsy result, meaning 92.4 percent true negative while 45 had B5a and B5b in the biopsy result, i.e. 7.6 percent false negative. This result was not changed when compared to the postoperative histopathology.

In the BI-RADS 4b Category 366 lesions had B2 and B3 results in the biopsy result, meaning 48.2 percent false positive while 393 had B5a and B5b in the biopsy result, i.e. 51.8 percent true positive. This result changed when compared to the postoperative histopathology because of the upgraded B3 lesions which were primary as BI-RADS 4b reported (5 cases), so the false positive decreased to 47.6 percent and the true positive increased to 52.4 percent.

In the BI-RADS 4c Category 12 lesions had B2 and B3 results in the biopsy result, meaning 8.8 percent false positive while 124 had B5a and B5b in the biopsy result, i.e. 91.2 percent true positive. This result was not changed when compared to the postoperative histopathology because none of the upgraded lesions was primary as BI-RADS 4c reported.

In the BI-RADS 5 Category 21 lesions had B2 and B3 results in the biopsy result, meaning 3.9 percent false positive while 516 had B5a and B5b in the biopsy result, i.e. 96.1 percent true positive.

This result changed when compared to the postoperative histopathology because of the upgrade B3 lesions which were primary as BI-RADS 5 reported (7 cases), so the false positive decreases to 2.6 percent and the true positive increased to 97.4 percent.

For the lesions which were assigned BI-RADS 4c (9 cases) and 5 (13 cases) with B2-category in the histopathology result, a re-biopsy was done for 2/9 cases in the BIRADS 4c and for 4/13 cases in the BIRADS 5 with no change of the histopathology result. A re-biopsy for 2/13 cases was recommended but it was declined from the patient with no further follow-ups in our records. Follow-ups for at least 12 months were done in 7/9 and 7/13 cases from both categories with no radiological change requiring re-biopsy.

## Discussion

Most previous studies originated from mammography screening programs, limited to an age range from 50–70 years. However, in our single-center study, the age ranged from16 to 96.5 years in all different categories.

In our study, B3 lesions comprised 4.5 percent of the total biopsies (n = 2,029). In one of the largest screening studies, El-Sayed et al. found that B3 lesions comprised 5 percent of all CNBs [14]. In agreement with El-Sayed et al., our underestimation rate of malignancy was 16.7 percent, compared to El-Sayed et al.’s 19.1 percent [14]. Of those, our underestimation rate of invasive lesions was 33.3 percent, compared to 37.7 percent [14], and our underestimation rate of non-invasive lesions was 66.7 percent, compared to 62.3 percent [14].

In agreement with Hoffmann et al., there was no significant difference between the B3 subgroups and malignant diagnosis after excision [5]. Timpe et al. also found that the overall underestimation rate of malignancy for B3 lesions was 24.3 percent, with a positive predictive value of 0.30 for atypical epithelial proliferation of ductal type (AEDPT). They also found an underestimation malignancy rate of 0.11 for papillary lesions, and no under-estimation malignancy rate for radial scar and flat epithelial atypia lesions [23].

Weigel et al. found that the positive predictive value was 0.28 for malignancy in the B3 category, 0.40 for atypical epithelial ductal proliferation (which was statistically significant compared to other subgroups), 0.08 for papillary lesions, and 0.20 for radial scars [24]. The statistical significance for atypical epithelial proliferation of ductal type (AEDPT) compared to other subgroups in this study, was due to the percentage of the AEDPT lesions from total B3 lesions; 51.6 percent (n = 47/91). In our study the atypical ductal hyperplasia was only 20 percent from all B3 lesions (n = 15/75). The frequency of papillary lesion in our cohort was 50.6 percent and 20 percent for atypical ductal hyperplasia, compared to El-Sayed et al.’s respective findings of 24 percent and 36 percent [25]. The difference in the order of the two most frequent lesions in our study is most probably because our cohort was not confined to a specific age group.

Our results regarding the B3 subcategories, may be different from some previous studies, but the positive predictive value for malignancy for the B3 category is very consistent and the positive predictive value for malignancy for ADH was the highest, although it was not statistically significant.

The B5a lesions comprised 5.6 percent from all biopsied lesions and 10.6 percent from all malignant lesions in our study. Weigel et al.’s screening program found that 82.3 percent (n = 5,082) of their patients had invasive breast cancers, 17.4 percent (n = 1,074) had DCIS, and 0.3 percent (n = 16) had LCIS [26]. In agreement with Weigel et al., regarding nuclear grade distribution [26], the percentage of high-grade, in-situ ductal carcinomas in our study was 65.2 percent, compared to 40.2 percent in Weigel et al. Moreover, 21.7 percent and 37.3 percent were intermediate grade in our study and Weigel et al.’s, respectively. Furthermore, 13.1 percent and 17.2 percent were low grade in our study and Weigel et al.’s, respectively.

Most ductal carcinoma in-situ lesions found at mammography present as microcalcifications, with approximately 75 percent of lesions presenting only as calcifications [27]. Up to 23 percent of DCIS may present as a mass or asymmetry, and approximately 12 percent are associated with a palpable abnormality [27, 28]. Our study showed that 75.6 percent of in-situ ductal carcinomas were biopsied under stereotactic-guidance and our results were consistent with previous literature.

There were several limitations to our study. First, we used two different mammography systems–digital luminescence mammography until 2010, and then flat panel mammography after 2010. Second, stereotactic guidance before 2011 was done using mammography, with patients seated. After 2011, we used a Mammotest table with patients in a prone position. In some cases, posteriorly located lesions can be challenging to biopsy in the prone position leading to technical failure. However, Wunderbaldinger found that there was no significant differences regarding the patient position in large core biopsy [29].

Third, different needle sizes (8-, 10-gauge) were used for VABs, and 14-gauge needles were used for CNBs. Previous studies found that malignancy underestimation rates for high-risk lesions and DCIS using 8-gauge needles ranged from 0 percent to 17 percent. However, rates ranged from 3 percent to 25 percent with 11-gauge needle [30–33]. This was consistent with our results shown in Table 3. Finally, ultrasound guidance for CNBs was done with three different ultrasound scanners. The latter two had a tissue harmonic imaging mode, providing sometimes the possibility for better visualization of the different lesions especially those with cystic or fatty parts.

## Conclusion

Our concordance rate for B3 (83.3 percent) and B5a (80.2 percent) lesions in the biopsies to postoperative histopathology matching to previously published literature. Surgical excision is our recommendation for lesions biopsied with a B3 category in the histopathology and a BIRADS category of (4b, 4c and 5). The PPVs for malignancy of B3 lesions showed no statistical significance between subgroups. Also, the nuclear grade of DCIS was not statistically significant in terms of upgrade into invasive breast cancer.
